# Mitochondria and the culture of the Borg

**DOI:** 10.1002/bies.201000073

**Published:** 2010-11

**Authors:** Emelie Braschi, Heidi M McBride, Understanding the integration of mitochondrial function within the reticulum, the cell, and the organism

**Affiliations:** University of Ottawa Heart InstituteOttawa, Canada

**Keywords:** fission, fusion, metabolism, mitochondrial dynamics, signaling

## Abstract

As endosymbionts, the mitochondria are unique among organelles. This review provides insights into mitochondrial behavior and introduces the idea of a unified collective, an interconnected reticulum reminiscent of the Borg, a fictional humanoid species from the Star Trek television series whereby decisions are made within their network (or “hive”), linked to signaling cascades that coordinate the cross-talk between mitochondrial and cellular processes (“subspace domain”). Similarly, mitochondrial dynamics are determined by two distinct processes, namely the local regulation of fission/fusion and the global control of their behavior through cellular signaling pathways. Indeed, decisions within the hive provide each mitochondrial unit with autonomous control of their own degradation, whereby mitochondrial fusion is inactivated and they become substrates for autophagy. Decisions within the subspace domain couple signaling pathways involved in the functional integration of mitochondria with complex cellular transitions, including developmental cues, mitosis, and apoptosis.

## The interconnected collective

Mitochondria evolved from a bacterial origin, as evidenced by the mitochondrial genomic sequences that have been retained throughout evolution 1. Using genetic evidence and ultrastructural resemblance as major arguments, Lynn Margulis (Sagan) published a theoretical paper in 1967 postulating that around 1–2 billion years ago, a proto-eukaryotic cell without mitochondria (anaerobic archaebacteria) captured an α-proteobacterium by endocytosis 2. One of the consequences of the endosymbiotic theory of mitochondria was the systemic conceptualization of these organelles as independent, rather incommunicative structures that primarily function as the energy powerhouse of the cell. Since most of the early metabolic experiments were performed on isolated mitochondria removed from their cellular milieu, scientists assumed that the primary determinant of metabolic rates was based upon the changes in the concentrations of metabolites. However, just as bacteria continue to surprise us with their ability to swarm and communicate within their colonies, the mitochondrial research community was equally surprised to learn that the mitochondria are not individual structures; rather they exist within an interconnected reticulum. Elegant studies in *Drosophila* and yeast initially revealed that the mitochondria are continually reshaped through ongoing fusion and fission events. These experiments unleashed a new paradigm in mitochondrial biology and have prompted a search for the meaning of the dynamic behavior of these organelles. Later work in both human and yeast systems led to the discovery of other proteins involved in the core fusion and fission machinery, thereby providing invaluable tools to manipulate the shape of the reticulum. Using these tools has permitted a greater understanding of the functional consequences of mitochondrial morphology.

However, the consequences of mitochondrial fusion and fission processes during the normal functioning of cells remain partly mysterious. Most certainly mitochondrial plasticity facilitates the movement and careful placement of these organelles within the cell. This is well illustrated in neurons, where mitochondrial delivery to the synapse requires the activation of signaling cascades to orchestrate the long range motility along microtubules, followed by the arrest and anchoring of mitochondria to the actin cytoskeleton 3, 4. It has also been shown that mitochondrial fission is a response to hyperglycemia 5, 6, and represents an essential aspect of mitochondrial quality control 7.

**Figure 1:**
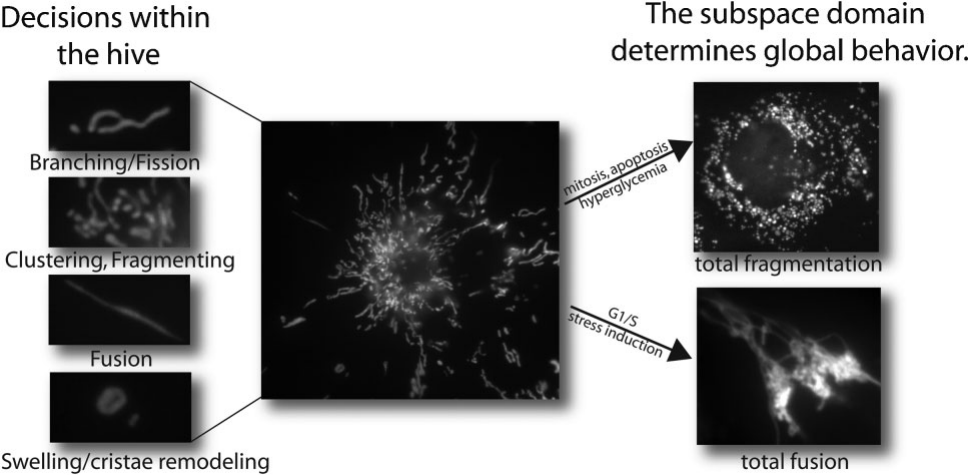
Controlling the mitochondrial collective. A single COS7 cell is illustrated in the center with the mitochondria labeled using a yellow fluorescent protein (shown in white). The steady state morphology of the mitochondria is continually remodeled based on local decisions within the “hive”. The four images in the left panel illustrate different dynamics, including branching, fission, clustering, fusion, and swelling. Changes in the cellular state, including cell cycle transitions, metabolic changes, stress, or cell death (right panels) lead to the activation of signaling pathways within the “subspace domain” that trigger global changes in the mitochondrial reticulum.

On the other hand, mitochondrial fusion is essential for the maintenance of mitochondrial DNA 4, 8, 9, therefore indirectly impacts on the rates of oxidative phosphorylation. The signaling cascades that coordinate the cross-talk between mitochondrial and cellular processes must now be identified, which in Borg terms would be analogous to the “subspace domain” that orchestrates their actions (Fig. 1). Indeed, recent years have seen an explosion in this area, with multiple signaling cascades emerging as key regulators of the core fission GTPase, Drp1, which has been shown to be SUMOylated 10, phosphorylated 11, ubiquitinated 12, and perhaps even S-nitrosylated 13, 14.

Evidence has also emerged that mitochondria may signal within their network (or “hive”) in a manner similar to that of a bacterial colony. This concept has not been widely considered within this context, but it is accepted that the coordinated activity of the reticulum involves lateral signaling cascades between the organelles. Functionally, this ensures metabolic synchrony. The most direct example of this is illustrated by the apoptotic waves of cytochrome c release and calcium flux during a death trigger 15, 16. Calcium waves are propagated between organelles and represent a prime example of lateral, inter-mitochondrial communication. Moreover, retrograde signals are sent from the mitochondria back to the nucleus, and have been the subject of a great deal of research, primarily in yeast model organisms 17. Retrograde signaling informs the nucleus of changing metabolic demands initiated at the mitochondria, leading to an up regulation of mitochondrial biogenesis 17–19.

Finally, it has been recently discovered by our laboratory that the mitochondria also send “pods” or vesicles to another intracellular organelle, the peroxisome 20, 21. Our ongoing research continues to reveal a much more communicative system of membrane transport than previously envisioned.

As the field of mitochondrial dynamics and signaling evolves, our knowledge of mitochondria, from a passive furnace that responds only to concentration gradients of metabolites into an organelle that functions as a signaling platform, is greatly expanding.

## Decisions within the hive

As recently proposed by Shirihai and coworkers 22, the mitochondria respond to two distinct processes, namely the local regulation of fission/fusion (within the hive) and the global control of their behavior through cellular signaling pathways (or within the subspace domain) (Fig. 1).

The local control of mitochondrial dynamics is not well understood, although it is known to be functionally essential 23–25. In the mammalian system there are three large GTPases that control mitochondrial fusion. The first two are outer membrane Mitofusins that have resulted from a gene duplication event (compared to yeast and lower organisms, which have only one Fzo1 GTPase to drive fusion). The third GTPase is an intermembrane space oligomeric protein called Opa1 that is required for both mitochondrial fusion and in the regulated assembly of the inner membrane cristae 26. The essential nature of mitochondrial fusion for metabolism and mtDNA stability was initially demonstrated more than 10 years ago in yeast model organisms 8, but has recently gained attention with the development of animal models lacking the fusion GTPases Mfn1 and/or Mfn2 23, 27, 28. Regardless of the tissue, the loss of Mfn2 leads to total atrophy and cell death, stemming from a systemic loss of mtDNA, accumulation of mtDNA mutations and metabolic incompetence. These results pose the question of how mitochondrial fusion could affect mitochondrial DNA integrity. Each cell can have hundreds of mitochondria, each containing many copies of the 16 kB plasmid mitochondrial DNA encoding tRNA, rRNA, and 13 proteins of the electron transport chain. The theory resulting from this work is that fusing the mitochondria will dilute any mutant mtDNA and allow the wild type genomes to contribute functional electron transport chain components 27, 28. Without fusion, these mutant genomes would accumulate within individual organelles, leading to their global dysfunction and presumably, autophagic clearance. This model is not without its caveats. The loss of Mitofusin2, for example, is believed primarily to block mitochondrial fusion, which is certainly well established. Moreover, Mfn2 is also a central component of the mitochondrial contact sites with the endoplasmic reticulum 29. Loss of these contacts will also seriously compromise calcium flux, which in turn may contribute to the loss of mitochondrial genomes. There is no certainty, however, whether it is mitochondrial fusion, the ER contacts, or even unknown functions of the mitofusins that are central to the loss of metabolism and genome stability. This discussion highlights the fact that much more work is required to understand how fusion modulates mitochondrial function.

In contrast to the many unknowns in mitochondrial fusion, examining the functional importance of mitochondrial fission has led to an unexpected new paradigm in mitochondrial quality control. In 2008 it was first shown that essentially all mitochondrial fission events lead to the depolarization of one of the “daughter” mitochondria and hyperpolarization of the other 7. This may be due to proton leakage during membrane scission, or some other physical aspect of the fission process. Importantly, the transient loss of potential may provide an opportunity to survey the metabolic health of the fragmented mitochondria. If they are actively respiring, they will regain their potential within a short time, and later fuse back into the reticulum. However, should they be respiration deficient, they would not regain potential. Consequently, there would be two consequences of organelles with low resting potential. Firstly, the protease Oma1 would become activated within the mitochondrial inner membrane and cleave the fusion GTPase Opa1, effectively exiling the depolarized organelle from the collective 30, 31. Secondly, the loss of potential would lead to the stabilization of the mitochondrial kinase PINK, and the recruitment of a ubiquitin E3 ligase called Parkin 32–36. Parkin recruitment leads to the delivery of the depolarized organelle to the autophagosome for degradation. Along the way, Parkin was also shown to ubiquitinate Mfn1 37, 38, providing a second hit in addition to the inactivation of Opa1, that would block re-fusion of this doomed organelle back into the reticulum. Together, this series of observations has been a breakthrough in our understanding of the function of mitochondrial fission in steady state quality control. Clinically, mutations in PINK1 and Parkin genes are causal for Parkinson's disease, strongly suggesting a common defect in mitochondrial quality control as an underlying feature of neurodegenerative disease. However, it is still unclear how the fission GTPase DRP1 (dynamin related protein 1) is initially recruited to the mitochondria, or how the fission site is chosen. Mathematical modeling of fission and fusion in the context of quality control would indicate that the process is likely stochastic in nature 39, where there is no theoretical need to actively identify dysfunctional regions of the reticulum. The ongoing, dynamic process of mixing and separating would eventually cull the damaged regions within the reticulum.

## The subspace domain

**Figure 2:**
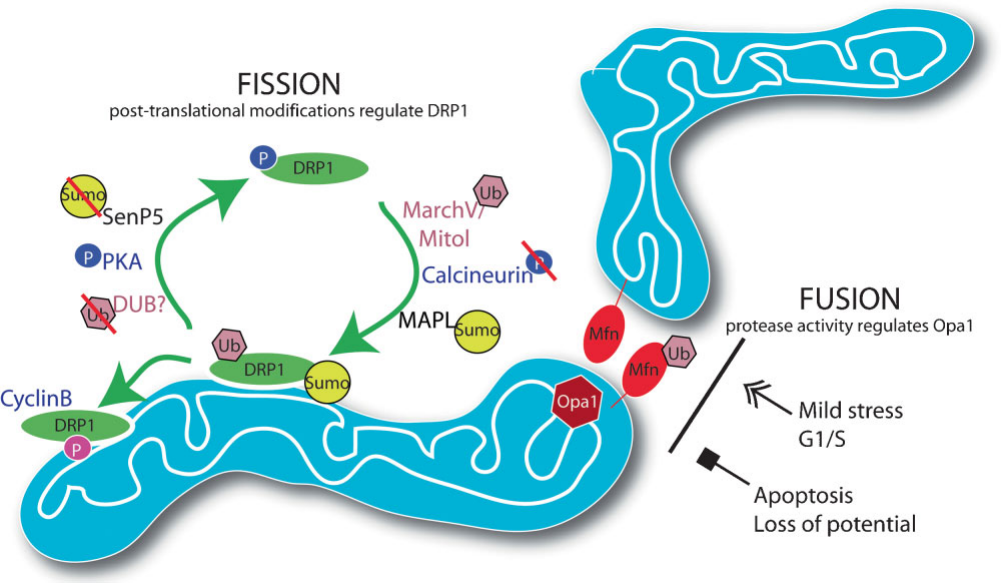
Signaling modules and switches that orchestrate mitochondrial behavior. This figure illustrates some of the complex post-translational modifications discussed within the text. Mitochondrial fission is achieved through the recruitment and oligomerization of the Dynamin Related Protein DRP1. There is evidence that this protein is SUMOylated, phosphorylated, and ubiquitinated, all of which will impact the activity of DRP1 in unique and overlapping ways. Modifying enzymes include MAPL, a SUMO E3 ligase, Protein kinase A, and cyclin B (kinases), and MarchV/Mitol (a ubiquitin E3 ligase). Deconjugating enzymes include SenP5 (a SUMO protease), calcineurin (a phosphatase), and potential deubiquitinating enzymes (DUBs) that have yet to be identified. Phosphorylation by PKA inhibits the recruitment of DRP1, while phosphorylation at a different site by Cyclin B promotes mitochondrial fission during mitosis. Mitochondrial fusion is regulated primarily through the proteolytic cleavage of the intermembrane space GTPase Opa1, and through the regulation of mitofusin (more clearly shown for yeast Fzo1) protein levels by ubiquitination. Mitochondrial fusion is inhibited during cell death and upon loss of electrochemical potential, and is stimulated in mild stress conditions and during G1/S. The combinatorial use of these modifications has not yet been tested directly in experimental models and there is much more to be discovered.

It has become most apparent that the level of interconnectivity within the mitochondrial reticulum changes between cell types and also during most cellular transition states. This makes it clear that the reticulum is highly responsive to the “subspace domain”, or the communication networks inherent in cell signaling paradigms. In fact, it has been the investigation of cell cycle, apoptosis, differentiation, and the stress responses that provided most information about the intersection between signaling cascades and changes in mitochondrial morphology and function. Most notable is the modulation of mitochondrial shape through the use of signal-driven posttranslational modifications by kinases, phosphatases, and ligases (Fig. 2).

### Ensuring transmission through the generations: Dynamics and the cell cycle

Global mitochondrial fragmentation is observed during mitosis, where it is thought to facilitate the segregation of the reticulum into daughter cells 40, 41, and during apoptosis, where the smaller fragments seem to accelerate the release of cytochrome c 42, 43. In contrast, the mitochondria become highly fused during the growth phase of G1 44, and in response to multiple forms of stress 45. The latter was shown in work by the Martinou group who observed hyperfused mitochondria as an early response to cellular stresses including UV irradiation (UV-C), actinomycin D, and cycloheximide. Mechanistically they revealed a critical role for a prohibitin-like protein Slp-2 to maintain Opa1 in a fusogenic form within the intermembrane space 45. It was speculated that fusion during stress would unify the reticulum to buffer them against damage and increase ATP production during this period, although the underlying mechanism has not been elucidated yet. It has been established that the mitochondria fuse into a highly interconnected network in G1/S 40, 41, 44, 46. More recently it was concluded that the hyperfusion of mitochondria was required for the buildup of cyclin E and entry into S phase 44. Consistent with this, the induction of fusion upon inhibition of Drp1 by the drug mDivi triggered entry into S phase, bypassing the requirement for growth factors 44. It is unclear whether the increased oxidative phosphorylation resulting from the fused network is sufficient to trigger S phase entry.

Alternatively, the fused reticulum could trigger downstream signaling events leading to S phase entry. If changes in mitochondrial dynamics are important for the regulation of the G1/S transition, this raises the possibility that cell-cycle regulators, in addition to controlling mitochondrial biogenesis, will also regulate the fusion or fission machineries. Possible candidates may be from the mTOR pathways that signal cell growth and proliferation. Indeed a number of proteins in this pathway have been localized to the mitochondria 47–49 and future work may uncover evidence of significant cross-talk between the transitions in mitochondrial dynamics, cell growth, and division.

There is also emerging evidence that the mitochondrial collective shifts into a fragmented state under diabetic conditions of high glucose levels 5, 50, 51. The molecular bases for this increased DRP1 activity, or the extent of inhibition of fusion are still unclear. Fragmentation also occurs during ischemia-reperfusion injury 52 and during cell death 53, where DRP1 recruitment is stabilized 54. Again, the molecular bases for this are not yet clear, although the dephosphorylation of DRP1 by calcineurin plays a role in at least some of these circumstances 55, 56. Importantly, the inhibition of DRP1-induced fragmentation has been shown to be protective in physiological models of disease, including Parkinson's disease 57, 58 and ischemia-reperfusion injury models 52, providing hope for future therapeutic development.

### Preparing to abandon ship: The collective kills the host

One of the earliest transitions in the subspace domain to signal the collective fragmentation of the mitochondria is the initiation of programmed cell death 42. Mitochondrial fragmentation clearly accompanies cell death, and inhibiting apoptotic fission delays the death program. However it is still unclear what the molecular purpose of the fission process really is. How do smaller mitochondria promote the formation of apoptotic pores that release the factors essential for cell death? Perhaps the fragmentation process is a side-reaction where the fission promoting machinery may have a secondary role in lipid organization, cristae remodeling and/or pore assembly 59–61. A great deal of interest continues to focus on obtaining answers to these questions.

Finally, there is a much broader question to consider when thinking about apoptosis. From the perspective of the unified collective, how did the mitochondria get involved in the apoptotic program in the first place? Considering the unique evolutionary history of the mitochondria, one wonders whether the control of apoptosis could have developed from their bacterial origin. Although the considerable phylogenetic distance makes it difficult to draw functional inferences from limited sequence similarities, the Bcl-2 family of proteins appears to be structurally related to bacterial pore-forming toxins, for example, colicins, and diphtheria toxin 62. In addition, components of the apoptotic machinery including VDAC, cyclophilin, cytochrome c, and the adenosine nucleotide carrier appear to be conserved in bacteria 63. It has been suggested that the replication advantage given to the pre-mitochondria may be related to the production of a long-lived toxin from which the bacteria-produced Bcl-2 “antidote” could rescue the host. Should the pre-mitochondria have been lost, then the longer-lived toxin would kill the host. This type of selection may have stabilized the host/parasite relationship and may represent the precursor stage to the mitochondria-dependent apoptotic machinery 64. Important to this model is the idea that one “rogue” mitochondrion must not attempt to kill the cell on its own, which may also explain the need for the reticulum to function in synchrony.

## Programming the subspace domain

### The role of SUMOylation in mitochondrial fission

This section will focus on our working hypothesis for some of the mechanisms that directly couple the subspace domain to changes within the mitochondrial hive. The most progress has been made in the identification of post-translational modifications of the fission GTPase DRP1. The first of these was the observation that DRP1 may be covalently conjugated to SUMO1, a process that was shown to stimulate mitochondrial fragmentation 10. The small ubiquitin-like modifier protein is similar to ubiquitin in the enzymology of conjugation, requiring an E1, E2, and E3 ligase to conjugate the ∼100 amino acid protein to a conserved lysine residue in the target 65. In 2004 SUMOylation was primarily thought to function within the nucleus, so the identification of a number of SUMO-immunoreactive proteins on the mitochondria was unexpected. The cycle of SUMO- and deSUMOylation is often used as a switch mechanism to initiate large protein complex assembly, or to disassemble them, as in the case of the septin GTPases 66–68. It is also used to reveal nuclear targeting signals and facilitate transport across the nuclear envelope. So what was the function of DRP1 SUMO conjugates? The collective evidence indicates that SUMOylation is not essential for mitochondrial fission, rather the cycle of SUMOylation and de-SUMOylation can promote mitochondrial fission, both in cell death 54 and at the G2/M transition 41. In this way, SUMOylation is a regulator of mitochondrial fission that activates the process during specific cellular transitions. This has been shown through the identification of the functional SUMO protease SenP5 69, and the characterization of the mitochondrial outer membrane SUMO E3 ligase, MAPL (Mitochondrial anchored protein ligase) 70, which has also been called MULAN 71 or GIDE 72. Experiments to reduce mitochondrial SUMOylation, either by overexpressing SenP5 or silencing MAPL, lead to a reduction in DRP1 SUMOylation, and in the case of SenP5 expression, increased mitochondrial connectivity. In contrast, experiments that increased mitochondrial SUMOylation, either by overexpressing MAPL, silencing SenP5, or overexpressing SUMO, lead to elevated mitochondrial fragmentation and higher levels of DRP1:SUMO1 conjugates 10, 69, 70.

Furthermore, these studies have shown that conditions of increased DRP1 SUMOylation stabilize the protein against degradation within solubilized cellular extracts, which may be indicative of a conformational shift. Together, these experiments have consistently shown that SUMOylation is a pro-fission process.

Mechanistically it has been more difficult to determine what exact step the DRP1 SUMOylation cycle may promote. DRP1 is a complex protein that undergoes a number of molecular transformations that are required to constrict and separate the mitochondrial tubule. These include its initial recruitment, the initiation of oligomer assembly, GTP hydrolysis, and membrane constriction, scission, and finally, oligomer disassembly 73. In the absence of the staged cell-free reconstitution of these steps in mammalian systems, one can only extrapolate on the existing biochemical and dynamic analysis of DRP1 function. Site directed mutagenesis studies done by the Feldman group have identified a number of potential sites within the middle “B domain” that were conjugated to SUMO1, SUMO2, and SUMO3 74. This domain is analogous to the PH domain of Dynamin 1 74. While mutations of all three sites rendered the protein SUMO-conjugation deficient, initial mitochondrial recruitment was still observed. This is consistent with our evidence that the loss of the mitochondrial SUMO E3 ligase MAPL did not significantly affect the overall morphology of the mitochondria in steady state 20. Our more recent work with the SUMO protease SenP5 has shown that just prior to the breakdown of the nuclear envelope during the G2/M transition, SenP5 translocates to the mitochondrial surface, where it accelerates the SUMO- and deSUMOylation cycle, promoting mitochondrial fragmentation 41. This was shown using photobleaching studies that revealed an increase in the rates of DRP1-YFP recycling on and off the mitochondrial membrane in mitosis compared to cells in interphase. A significant increase in the oligomeric forms of DRP1 in mitotic cells, consistent with a higher fraction of functional fission sites during this time, was also observed.

Concomitantly, there was a decrease in total mitochondrial (and DRP1) SUMOylation when SenP5 was delivered to the membrane during mitosis. Functionally, the silencing of SenP5 led to a cell cycle arrest at G2/M, suggesting that its delivery to the mitochondria may be an important cell cycle checkpoint, although the loss of deSUMOylation activity within the nucleus could also have been responsible for this arrest 41.

In contrast to the acceleration of DRP1 recycling during mitosis, apoptotic fragmentation, which is considered irreversible, led to the Bax/Bak-dependent stable SUMOylation of DRP1 on the mitochondrial membranes, which coincided with a complete loss in recycling off the mitochondrial membrane 54. The block in DRP1 recycling and stable SUMOylation occurred after mitochondrial fragmentation was completed, suggesting a potential role for deSUMOylation in the disassembly of DRP1 oligomers. Our combined studies in cell cycle and cell death have led to the development of a working model, proposing that the initial recruitment of DRP1 may be primarily based on lipid binding affinities, akin to the PH domain of Dynamin 1. DRP1 SUMOylation may alter its conformation in order to enhance the retention of DRP1 on the membrane and association with the downstream partners like Fis1 and Mff. As an outer membrane protein, the SUMO E3 ligase MAPL could SUMOylate DRP1 following recruitment.

Given the precedents already set for other SUMO substrates, it is likely that the SUMOylated form of DRP1 is very transient, with deSUMOylation following quickly as part of the process of conformational change. During mitosis, this entire cycle would be enhanced by the delivery of the SUMO protease. In contrast, during cell death, SUMOylation leads to a post-fission form of DRP1 that is trapped on the mitochondrial membrane. This may be consistent with the increase in SUMOylation observed in the GTP binding mutant DRP1/K38A 74, where following GTP hydrolysis, SUMOylation may occur as a mechanism to disassemble the oligomers following constriction. The increased SUMOylation of this mutant would also suggest that MAPL preferentially recognizes the empty or GDP-bound forms of DRP1, which would be consistent with the SUMOylation of the initially recruited protein, as well as a role for the SUMOylation cycle to diassemble the DRP1 oligomer post-fission. In this way SUMOylation is envisioned as playing a “chaperone-like” function in the transitioning of DRP1 into and out of the oligomers.

### Additional DRP1 modifications couple fission to the cellular state

Subsequent to the identification of DRP1 SUMOylation, it has been shown that DRP1 also undergoes a number of regulated phosphorylation events (Fig. 2). It was first demonstrated that DRP1 Ser585 is phosphorylated by Cdk1/CyclinB upon the onset of mitosis, a modification that was shown to increase its recruitment and drive mitochondrial fission for efficient segregation into the two daughter cells 40. Since then, DRP1 was also shown to be phosphorylated by protein kinase A (PKA) at Ser637, which inactivates the GTPase, leading to an inhibition of mitochondrial fission and protection against cell death 11, 75. On the other hand, increased Ca^2+^ concentrations activate the phosphatase calcineurin which dephosphorylates DRP1/Ser637 thereby restoring DRP1 recruitment to the mitochondria, leading to increased fragmentation 11, 56. The recent use of a peptide inhibitor of calcineurin has extended these studies, showing how the retention of DRP1 in the phosphorylated state during cell death was highly protective 55. Importantly, it is possible that, *in vivo*, phosphorylation events would be coupled to a second post-translational modification, which could influence GTPase or oligomer assembly. For example, the phosphorylation of Ser637 of Drp1 by PKA was first shown to inhibit DRP1 function 11, 75. However, phosphorylation of this same serine residue by the Ca^2+^/Calmodulin-dependent protein Kinase I α (CaMKIα) was shown to increase the translocation of Drp1 to the mitochondria, and to trigger mitochondrial fragmentation in neurons 76. Interestingly, this last study examined the neuron specific Drp1, which lacks a region of the Variable Domain. This domain is alternatively spliced in different Drp1 isoforms.

Interestingly, the spliced domain contains SUMOylation sites 74. Hence, depending on the SUMOylation state of Drp1, the phosphorylation of DRP1 on Ser637 would either increase or decrease fission rates. These examples highlight the complexity of understanding the regulation of Drp1 since the consequences of a specific post-translational modification may vary depending on the cellular context and on the presence of multiple post-translational modifications. Ubiquitination may also play a role in the regulation of DRP1 activity 12, 77–79. The downregulation of a mitochondrial-anchored ubiquitin E3 ligase Membrane Associated Ring Finger 5 (MARCHV) promotes the recruitment of Drp1 to the mitochondria 12, 79. However, FRAP studies have demonstrated that the enrichments formed by DRP1 are not as dynamic, which leads to a decrease in mitochondrial fission and to a reticular phenotype. It is therefore possible that the ubiquitination of DRP1 is also a requisite for the formation of active oligomers post-recruitment 12. This would indicate a requirement for a specific mitochondrial deubiquitination enzyme (DUB) for DRP1, which has not yet been identified. More recent work with MARCHV has also shown that Mfn1 is a likely substrate, since Mfn1 protein levels are increased upon silencing of MARCHV, which contributes to the fused phenotype 79. This is reminiscent of the function of the yeast Skp-Cullin ubiquitin E3 ligase Mdm30, which regulates the turnover of the yeast orthologue of the human mitofusins, Fzo1 80–82. Therefore ubiquitin E3 ligases are also acting on the fission/fusion machinery in order to regulate their activity, by promoting degradation and also through non-proteasomal pathways.

## Glitches in the collective: The path to disease

The smooth functioning of the mitochondrial reticulum appears to depend entirely upon an ability to respond to global and local cues in order to shift morphology and position. It has become increasingly evident that a number of human diseases may be directly caused by the disruption of the mitochondrial reticulum 83. The most obvious links to disease are neurodegenerative conditions where proteins of the morphology machinery are mutated, most notably Charcot-Marie Tooth Type 2A and Dominant Optical Atrophy 83. However more common neurodegenerative diseases including Alzheimer's 13, 84, Huntington's 85, 86, and Parkinson's disease 87 are also directly linked to mitochondrial dysfunction. These diseases, as with the ischemic conditions associated with heart disease and stroke 52, may stem from a reduction in mitochondrial quality control pathways, which, as discussed above, are acutely dependent upon ongoing plasticity within the network. Mitochondria have also made recent, unexpected appearances in many laboratories interested in immunology and septic shock. The first example was the identification of a core component required to signal viral infection and activate the immune response. An outer mitochondrial membrane protein called MAVS functions as the “launchpad” for the Nf-kB and type I IFN antiviral transcriptional response 88–91. Why this immune complex must be localized to the mitochondria is not yet clear, although the proximity to the apoptotic machinery is unlikely to be a coincidence. The goal of the infected cell is to stave off death until it is able to alert the neighboring cells of the viral invasion, so placing the signaling pathway that releases interferons within the apoptotic organelle may allow the temporary inhibition of cell death 92. Consistent with this, a number of recent publications have also linked MAVS activity to the activation of mitochondrial fusion 93, 94, which is known to be protective against cell death 43.

Finally, the evolutionary origins of the mitochondrion as a potential pathogen have emerged once again in a recent study with a great deal of relevance to emergency room physicians 95. It has been long known that various bone fractures or trauma injuries lead to a toxic shock response that utilizes the same molecular pathways as those engaged in severe bacterial infections, although the reason for this was unknown. It has now become clear that mitochondria released from the ruptured cells are the culprit 96. It appears that their signature mitochondrial DNA and the unique formylated peptides translated by their own mitochondrial ribosomes are recognized as the bacteria that they once were. These elements of the released mitochondria were shown to directly activate the Toll-like receptors on immune cells within the circulation, prompting the critically dangerous shock response. In this case, those mitochondria liberated from the “collective” bind to the receptors of potential host cells, which in an odd turn of events, takes them back to earlier times, even if it was about a billion years ago.

## Conclusions

Much has been written on the importance of mitochondrial dynamics in cellular function.

This essay has focused on the concepts that have emerged to explain the responsiveness of the mitochondria to both global and local cues. There are many holes that remain in our understanding of the signaling pathways that activate mitochondrial fusion or fission, meaning that we do not yet comprehend the “subspace domain” to which the mitochondria are intimately coupled. What is known is that, much to our initial surprise, the mitochondria function as a highly orchestrated reticulum within cells. This, combined with the fact that the mitochondria have acquired most of their proteins from the host to become a truly hybrid organism, have led to a responsive and effective organelle. The Borg of Star Trek are an interconnected collective focused on the assimilation of other life forms in order to achieve perfection. In the case of the mitochondria, it must be concluded that the culture of the Borg will ultimately benefit us all.
